# Linker-Engineered Tyrosine–Azide Coatings for Stable Strain-Promoted Azide–Alkyne Cycloaddition (SPAAC) Functionalization

**DOI:** 10.3390/polym17222969

**Published:** 2025-11-07

**Authors:** Suho Park, Himani Bisht, Jiwoo Park, Seongchul Park, Yubin Hong, Daeun Chu, Minseob Koh, Hojae Lee, Daewha Hong

**Affiliations:** 1 Department of Chemistry and Chemistry, Institute for Functional Materials, Pusan National University, Busan 46241, Republic of Korea; sohomish@pusan.ac.kr (S.P.); himani@pusan.ac.kr (H.B.); minseob.koh@pusan.ac.kr (M.K.); 2 Institute for Future Earth, Pusan National University, Busan 46241, Republic of Korea; 3 Department of Chemistry, Hallym University, Chuncheon 24252, Republic of Korea

**Keywords:** strain-promoted azide–alkyne cycloaddition (SPAAC), melanin-inspired coatings, tyrosine–azide derivatives, metal-free clickable coatings, soft lithography

## Abstract

Strain-promoted azide–alkyne cycloaddition (SPAAC) is widely used in solution-phase bioconjugation. However, its application in surface chemistry remains limited because substrate-independent azide films that remain stable upon reaction with bulky strained alkynes have not yet been developed. In this study, we address this challenge using a melanin-inspired coating based on tyrosine–azide derivatives with different linkers. In particular, we investigated how differences in linker length and hydrophilicity affect the hydrophobic interactions within the film network and, ultimately, determine film stability. Specifically, Tyr-3-N_3_, a tyrosine–azide derivative having an azide group tethered to tyrosine through a short three-carbon alkyl linker, was identified as optimal, forming azide-presenting films via tyrosinase-mediated oxidation and retaining integrity during SPAAC with external dibenzocyclooctyne (DBCO) ligands. The optimized poly(Tyr-3-N_3_) coatings enabled efficient methoxypolyethylene glycol (mPEG) immobilization, thereby exhibiting excellent antifouling performance against protein adsorption, and further supported spatially controlled protein patterning through soft lithography techniques such as micromolding in capillaries (MIMIC) and microcontact printing (µCP). The approach was broadly applicable with a range of inorganic and polymeric substrates, as well as living cell surfaces; even after encapsulation and SPAAC-based functionalization, the cells remained viable. Collectively, these findings establish a substrate-independent and biocompatible coating platform that preserves film stability through SPAAC functionalization, supporting applications in antifouling coatings, biosensing, and cell surface engineering.

## 1. Introduction

Strain-promoted azide–alkyne cycloaddition (SPAAC) enables fast, metal-free, bioorthogonal ligation in water using cyclooctynes such as difluorinated cyclooctyne (DIFO) [1], dibenzocyclooctyne (DIBO) [2], aza-dibenzocyclooctyne (DBCO) [3], biarylazacyclooctynone (BARAC) [4], and bicyclo [6.1.0] non-4-yne (BCN) [5]. In bioconjugation, SPAAC enables modular attachment of fluorophores [1,2], polymers [3,6], and site-specific tagging of proteins and antibodies through genetic code expansion to introduce azide handles [7]. They are also widely used to functionalize and coat surfaces [8], modify nanomaterials [9], and form hydrogels under metal-free conditions compatible with complex biological environments [10,11].

In surface chemistry, the immobilization of functional molecules on surfaces via SPAAC represents a related but distinct approach, which involves reactions between freely diffusing (bio)molecules in solution. Surface immobilization occurs at the interface between a solid substrate and water-soluble functional molecules, thus offering valuable tools for achieving antifouling [12], biosensing [13], and cell surface engineering [14]. Ideally, these coatings should rely on universally applicable and biocompatible azide films that can be formed on diverse substrates, support rapid surface SPAAC conjugation, and maintain a stable film network throughout the process. However, meeting all these requirements simultaneously presents a significant technical challenge.

Recently, melanin-inspired coating strategies based on tyrosine derivatives have emerged as a promising route to meet these stringent requirements [15]. As next-generation alternatives to polydopamine [16], melanin-inspired coatings enabled film formation exclusively in the presence of tyrosinase under mild aqueous conditions and can be used with diverse substrates, including living cell surfaces [17]. In previous studies, we demonstrated the versatility of this platform by synthesizing various tyrosine derivatives and applying them to the construction of functionalizable films [17], initiator films for subsequent polymerization [18], and antifouling coatings [19,20]. Apart from its role in melanin-inspired film formation, the tyrosine moiety itself can also serve as a useful tool for bioconjugation, as it can be modified using hypervalent iodine reagents [21] or 4-phenyl-1,2,4-triazoline-3,5-dione [22], or through deoxyribozyme-catalyzed reactions with 2′-azido-2′-deoxyadenosine 5′-triphosphate [23].

Refocusing on the melanin-mimicking aspect, we previously designed tyrosine–tri(ethylene glycol)–azide (Tyr–(EG)_3_–N_3_) [24]. In this molecule, the azide group is tethered to tyrosine through an (EG)_3_ linker and can undergo tyrosinase-mediated oxidation to form azide-presenting films, which subsequently enable the copper-catalyzed azide–alkyne cycloaddition (CuAAC)-based immobilization of relatively small terminal alkyne–functionalized ligands without noticeable disruption of the film network. Building on this result, we focused on SPAAC as a copper-free alternative suitable for biocompatible applications. However, unlike in homogeneous solution-phase SPAAC, interfacial reactions at azide-coated surfaces raise concerns regarding film stability. Melanin-mimetic coatings assembled through noncovalent and covalent interactions are intrinsically vulnerable to harsh environments, and the rapid kinetics and steric bulk of strained cyclic alkynes are expected to exacerbate this instability during functionalization [25,26]. Indeed, in our previous work, we observed that SPAAC reactions with films derived from Tyr–(EG)_3_–N_3_ undergo film degradation rather than the stable immobilization of ligands, limiting their applicability for copper-free surface functionalization. To address these issues, we aimed to synthesize a series of tyrosine–azide derivatives with various linkers differing in length and hydrophilicity, thereby enabling us to tune film stability and ultimately identify an optimized precursor for efficient SPAAC-based surface functionalization while preserving film integrity.

## 2. Materials and Methods

### 2.1. Materials

Tyrosinase from mushrooms (≥1000 units mg^–1^, Sigma-Aldrich, Burlington, MA, USA), DBCO acid (≥95%, Sigma-Aldrich), methoxypolyethylene glycol (mPEG) thiol (mPEG-thiol, Mn 2000, Sigma-Aldrich), DBCO-mPEG (Mn 2000, Broad Pharm, San Diego, CA, USA), DBCO-EG_4_-biotin (99%, Broad Pharm), isothiocyanate conjugated albumin (FITC-albumin, Sigma-Aldrich), Alexa Fluor^TM^ 488 streptavidin (Invitrogen, Waltham, MA, USA), Alexa Fluor^TM^ 647 streptavidin (Invitrogen), dopamine hydrochloride (≥98.0%, Sigma-Aldrich), phosphate-buffered saline (PBS, Thermo Fisher, Waltham, MA, USA), trizma^®^ hydrochloride (≥99%, Sigma-Aldrich), trizma^®^ base (≥99%, Sigma-Aldrich), potassium phosphate dibasic (≥98.0%, Sigma-Aldrich), and potassium phosphate monobasic (≥99.0%, Sigma-Aldrich) were used as received. Organic solvents, including acetone (≥99.5%), isopropyl alcohol (*i*-PrOH, ≥99.5%), methanol (MeOH, >99.9%), and ethanol (EtOH, 94.5%) were purchased from Daejung (Siheung, Republic of Korea). Deionized water was purified using a Millipore water purification system. Silicon wafers were purchased from the National Nanofab Center (Daejeon, Republic of Korea). A titanium dioxide (TiO_2_) wafer was prepared by depositing 100 nm Ti onto a silicon wafer, with the outer layer surfaces allowed to naturally oxidize under ambient conditions. A gold surface was prepared by depositing 100 nm Au over a 20 nm Ti ad-layer onto a silicon wafer. Other substrates included indium tin oxide (ITO, Daihan Scientific Co., Wonju, Republic of Korea), glass (Marienfeld, Lauda-Königshofen, Germany), polystyrene (PS, SPL), polytetrafluoroethylene (PTFE, Daedeok Science, Daejeon, Republic of Korea), polyethylene terephthalate (PET, Graphene Square, Seoul, Republic of Korea), polyoxymethylene copolymer (POMC, Goodfellow, Huntingdon, UK), acrylic (Q-Tech, Incheon, Republic of Korea), and nylon (Goodfellow). A cyclic olefin copolymer (COC) substrate was sourced from the Electronics and Telecommunications Research Institute (Daejeon, Republic of Korea).

### 2.2. Surface Coating with Tyrosine–Azide Derivatives on Planar Surfaces

A tyrosinase stock solution was prepared by dissolving 50 kU of lyophilized enzyme in 5 mL of 50 mM potassium phosphate buffer (50 mM, pH 6.5). The solution was aliquoted (200 µL, 10 kU mL^−1^) and stored at 4 °C. Substrates (1.4 cm × 1.4 cm) were cut to size and washed sequentially with acetone, *i*-PrOH, EtOH, and deionized water; polymeric substrates received an additional rinse with MeOH before the final deionized-water wash. Coating precursors (e.g., Tyr-3-N_3_, 2 mM in PBS) were placed in a reaction chamber (5 mL; 3.5 cm diameter × 1.75 cm height). Tyrosinase (20 µL of the 10 kU mL^−1^ stock) was added, and the chamber was incubated at 30 °C with shaking at 110 rpm for 6 h to generate poly(Tyr-3-N_3_) films. After incubation, the substrates were removed, rinsed with deionized water, and air-dried. For comparison, poly(Tyr-1-N_3_) and poly(Tyr-2-N_3_) coatings were prepared under identical conditions. As a control, polydopamine films were obtained by immersing substrates in a dopamine hydrochloride solution (2 mg mL^−1^ in 10 mM Tris buffer, pH 8.5) using the same incubation parameters. For surface functionalization, poly(Tyr-3-N_3_)-coated substrates were immersed in a 1 mM DBCO-ligand solution (PBS) for 30 min. Polydopamine-coated substrates were treated with a 1 mM thiol-ligand solution (PBS) for the same duration. All substrates were subsequently rinsed with PBS and deionized water to remove unbound species.

### 2.3. Micromolding in Capillaries (MIMIC)

Albumin line patterns were fabricated on the poly(Tyr-3-N_3_)-coated surfaces using the MIMIC technique with PDMS microchannels prepared from Sylgard 184 (Dow Corning, Midland, MI, USA), as described previously [27]. The PDMS channels (50 µm width × 100 µm spacing × 70 µm height) were rendered hydrophilic by exposure to oxygen plasma (Femto Science, Hwaseong, Republic of Korea) for 5 min and then placed on the poly(Tyr-3-N_3_)-coated substrates. A DBCO-mPEG solution (20 µL of 1 mM in PBS) was introduced into the channels for 10 min. The PDMS stamp was then gently peeled off while a continuous PBS rinse was applied, and the substrate was washed with PBS. The patterned substrate was immersed in PBS for an additional 10 min, followed by incubation in FITC-albumin (1 mg mL^−1^ in PBS) for 1 h at room temperature (RT) in the dark. After a final PBS wash, green fluorescent protein line patterns, which were produced by regioselective adhesion of FITC-albumin, were visualized with a Leica DM3000 (Leica Microsystems, Wetzlar, Germany) fluorescence microscope. As a control, the same procedure applied to polydopamine-coated surfaces failed to generate line patterns because of the lack of SPAAC reactivity.

### 2.4. Microcontact Printing (µCP)

Streptavidin grid patterns were generated by µCP using PDMS stamps bearing grid features (100-µm width × 50-µm spacing). Stamps were rendered hydrophilic by oxygen plasma treatment for 5 min, then inked with 5 mM DBCO-EG_4_-biotin in EtOH (50 µL) and dried gently with a distant stream of air. The inked stamp was brought into conformal contact with the azide-presenting poly(Tyr-3-N_3_)-coated substrates, a 100 g weight was placed on top for 10 min, and the stamp was removed by vertical peel-off. The substrates were rinsed with flowing EtOH for 1 min, dried, sonicated in deionized water for 1 min to minimize nonspecific adsorption, and dried again. To passivate the remaining surface azides, the patterned substrates were incubated in 1 mM DBCO-mPEG in PBS for 30 min, then rinsed with deionized water and dried. The substrates were immersed in PBS for 10 min, followed by incubation in Alexa Fluor^TM^ 488 streptavidin (50 µg mL^−1^ in PBS) for 30 min at RT in the dark. After a final PBS wash, green, fluorescent streptavidin grid patterns were observed using the Leica DM3000 fluorescence microscope. As a control, the identical protocol on polydopamine-coated surfaces did not produce grid patterns because of the absence of SPAAC reactivity.

## 3. Results

**Figure 1:**
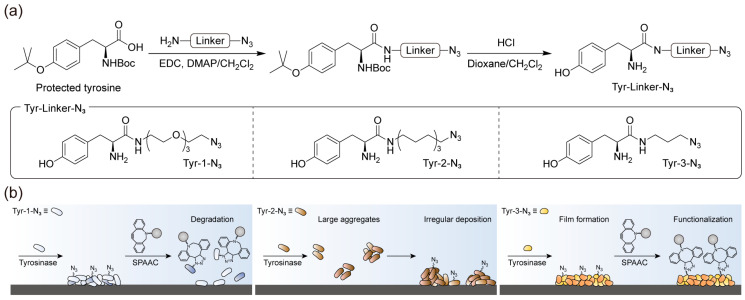
Schematic of the linker-screening strategy using tyrosine–azide derivatives to optimize film stability and enable SPAAC functionalization. (**a**) Common synthetic route from protected tyrosine to tyrosine–azide derivatives, yielding Tyr-1-N_3_ with an (EG)_3_ linker, Tyr-2-N_3_ with a 10-alkyl linker, and Tyr-3-N_3_ with a 3-alkyl linker. (**b**) Conceptual depiction of how the initial linker structure influences hydrophobic interactions within the deposited film, producing differences in film stability during subsequent SPAAC reactions with bulky strained alkynes.

Figure 1 illustrates the overall concept of this study, which aims to construct a stable azide-presenting film that can be functionalized with external ligands via SPAAC. As shown in Figure 1a, a common synthetic strategy was employed, in which the carboxylic acid group of the protected tyrosine was coupled with amine bearing an azide group through different linkers, followed by deprotection (See the Supplementary Materials for the detailed synthetic process). This approach yielded a molecular framework comprising two key functionalities: a phenolic amine, which served as a universal dormant coating precursor activated by tyrosinase, and an azide group, which was exposed on the film surface for subsequent SPAAC functionalization. Using this framework, three representative derivatives were synthesized: Tyr-1-N_3_, containing an (EG)_3_ linker; Tyr-2-N_3_, containing a 10-alkyl linker of comparable length; and Tyr-3-N_3_, containing a shorter 3-alkyl linker (Figure 1a). We designed this molecular system based on the hypothesis that the coating formed on the surface would be inherently water-insoluble, originating from the assembly of oxidative adducts generated under aqueous conditions (Figure 1b) [28]. Within this assembled network, hydrophobic interactions are assumed to play an important role in maintaining the compactness and stability of the film. Although the film itself does not dissolve in water, the extent of these hydrophobic interactions can vary depending on the linker structure of the precursor. When bulky strained alkynes used in SPAAC reactions are introduced in aqueous media, their incorporation may locally disturb the existing hydrophobic packing, which could lead to partial weakening of the film. Therefore, reinforcing or tuning these intra-film hydrophobic interactions prior to the click reaction is expected to help preserve the structural integrity of the coating after SPAAC. The adducts generated during film formation exhibit linker-dependent solubility but gradually become insoluble through sequential tyrosinase-catalyzed oxidation reactions.

As an initial assessment, the solubility of the precursors in PBS (1 mM, 5 mL) and their film-formation behavior on gold under tyrosinase-induced oxidation were investigated (Figure S1). Visual inspection of the initial dissolved state revealed that Tyr-1-N_3_ and Tyr-3-N_3_ were highly soluble, yielding transparent solutions, whereas Tyr-2-N_3_ showed poor initial solubility, as well as turbidity, likely because of its long hydrocarbon chain (Figure S1a). After the addition of 20 µL tyrosinase stock solution (10 kU mL^−1^) and subsequent oxidation for 6 h, all solutions became less transparent: in detail, Tyr-1-N_3_ and Tyr-3-N_3_ remained relatively well-dispersed, whereas Tyr-2-N_3_ exhibited severe aggregation, large visible particles, and phase separation, indicating a poor candidate for uniform film formation (Figure S1a). Consistent with this observation, ellipsometry measurements showed that films derived from Tyr-1-N_3_ and Tyr-3-N_3_ exhibited relatively small standard deviations in thickness across substrates, whereas those from Tyr-2-N_3_ showed much larger deviations owing to heterogeneous deposition (Figure S1b). These differences can be attributed to the better initial solubility of Tyr-1-N_3_ and Tyr-3-N_3_, aided by the hydrophilic (EG)_3_ linker in Tyr-1-N_3_ and the shorter hydrophobic linker in Tyr-3-N_3_, unlike the poor solubility and resulting aggregation of Tyr-2-N_3_ in the aqueous medium.

**Figure 2:**
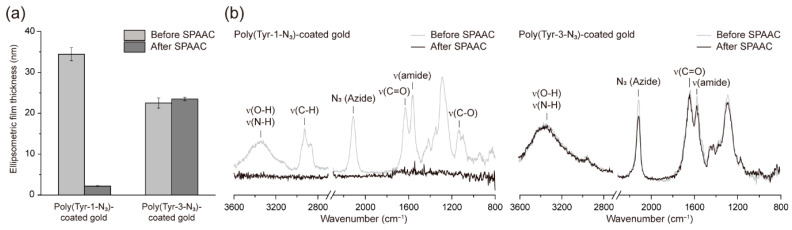
Characterization of poly(Tyr-1-N_3_) and poly(Tyr-3-N_3_) films before and after SPAAC functionalization with DBCO acid. (**a**) Ellipsometric thickness measurements showing a dramatic decrease for poly(Tyr-1-N_3_) films after SPAAC, whereas no thickness loss was observed for poly(Tyr-3-N_3_) films. (**b**) ATR-FTIR spectra of coated gold before and after SPAAC; poly(Tyr-1-N_3_) films exhibit attenuation of most characteristic peaks, while poly(Tyr-3-N_3_) films retain their features with only a reduced azide-peak intensity.

Subsequently, we investigated the stability of the films formed from Tyr-1-N_3_ and Tyr-3-N_3_ upon SPAAC functionalization, denoted poly(Tyr-1-N_3_) and poly(Tyr-3-N_3_), respectively (Figure 2). Among various SPAAC reagents such as DIFO, DIBO, DBCO, BARAC, and BCN, DBCO was selected owing to its wide commercial availability and high reactivity under mild aqueous conditions [29]. For film-stability testing, we specifically employed DBCO acid (1 mM in PBS) as a small-molecule probe, because it lacks thiol and amine functionalities that could otherwise engage in side reactions with polydopamine-like melanin moieties [30]. This choice ensures that the observed functionalization arises solely from the SPAAC reaction between surface azides and DBCO. Ellipsometric measurements revealed distinct differences between the two films (Figure 2a): the thickness of poly(Tyr-1-N_3_) decreased dramatically from 34 to 2 nm after reaction with DBCO acid, indicating severe film degradation, whereas poly(Tyr-3-N_3_) remained essentially unchanged (22 to 23 nm), indicating stable functionalization. To verify these results, attenuated total reflection Fourier transform infrared (ATR-FTIR) spectra were analyzed before and after SPAAC (Figure 2b). Poly(Tyr-1-N_3_)-coated gold exhibited broad absorption bands at 3500–3200 cm^−1^ (ν(O–H), ν(N–H)), 1629 cm^−1^ (ν(C=O)), and 1564 cm^−1^ (ν(amide)), along with additional peaks at 2927 cm^−1^ (ν(C–H)) and 1133 cm^−1^ (ν(C–O)), characteristic of EG units [31], as well as a strong azide peak at 2112 cm^−1^ [32]. Following SPAAC with DBCO, the intensities of all these peaks decreased markedly, which is consistent with the ellipsometry results of film degradation. In contrast, poly(Tyr-3-N_3_)-coated gold displayed a similar spectrum but without the 1133 cm^−1^ (ν(C–O)) band because of the absence of EG units, and upon SPAAC functionalization, only the azide peak decreased whereas other characteristic peaks remained largely unchanged. This result confirms that, unlike poly(Tyr-1-N_3_), the poly(Tyr-3-N_3_) film remains intact after SPAAC while successfully consuming the surface azides for functionalization. This difference is likely a result of differences in the hydrophobic character of the oxidative adducts that assemble the films into water-insoluble networks. Although both films were insoluble before reaction, the poly(Tyr-1-N_3_) network containing the hydrophilic (EG)_3_ linker relied more on the weaker hydrophobic cohesion. During the rapid interfacial SPAAC with bulky DBCO, these vulnerable interactions were disrupted, leading to partial disassembly and material loss into the surrounding aqueous phase. In contrast, the poly(Tyr-3-N_3_) network, built from a shorter alkyl linker, exhibited stronger hydrophobic interactions and, thus, retained its integrity under the same conditions. As a control, the same experiment was conducted using the poly(Tyr-2-N_3_) film (Figure S2), where no noticeable decrease in thickness was observed after the SPAAC reaction (Figure S2a), and ATR-FTIR analysis confirmed that the reaction proceeded through a reduction of the azide peak without any significant changes in other characteristic peaks (Figure S2b). However, as mentioned earlier, the film exhibited large standard deviations in thickness and was therefore not selected as a suitable or optimized coating platform. Using the optimized poly(Tyr-3-N_3_) film, we subsequently employed two types of functional DBCO derivatives to modulate surface properties via the surface SPAAC reaction.

First, we employed DBCO-mPEG to construct antifouling surfaces against fibrinogen adsorption. Conventional polydopamine coatings allow amine or thiol conjugation via oxidized quinone groups [30], and mPEG immobilization on these films has been reported [16]. However, these approaches require alkaline conditions (pH 8.5) to oxidize catechols to reactive quinones to a limited extent and involve long reaction times (approximately 3 h). In contrast, SPAAC on poly(Tyr-3-N_3_) is expected to enable rapid mPEG conjugation in a physiological buffer (pH 7.4, 30 min). After mPEG attachment, the film thickness increased from 30 to 33 nm, whereas the water contact angle decreased from 40° to 35°, confirming measurable surface modification. The resulting mPEG-modified coating is hereafter referred to as poly(Tyr-3-N_3_)-mPEG. The antifouling performance of the resulting poly(Tyr-3-N_3_)-mPEG surface was then evaluated using fibrinogen adsorption quantified by enzyme-linked immunosorbent assay (ELISA) (Figure S3) [33]. Compared with an uncoated gold surface, the poly(Tyr-3-N_3_)-mPEG surface exhibited nearly complete resistance to fibrinogen adsorption (3%; Figure S3a). In contrast, mPEG-thiol immobilization on polydopamine films under the same conditions resulted in negligible suppression of fibrinogen binding (Figure S3b). This result shows that antifouling performance can be achieved under physiological conditions within 30 min using SPAAC on poly(Tyr-3-N_3_), unlike conventional polydopamine coatings.

**Figure 3:**
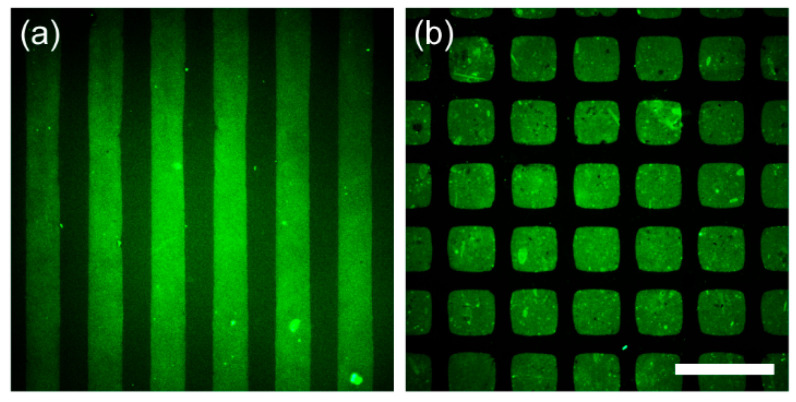
Fluorescence-microscopy images of protein patterning on poly(Tyr-3-N_3_) films via soft lithography. (**a**) Fluorescence image obtained after MIMIC with DBCO-mPEG and subsequent exposure to FITC-albumin. (**b**) Fluorescence image obtained after µCP with DBCO-EG_4_-biotin stamping, DBCO-mPEG passivation, and Alexa Fluor^TM^ 488 streptavidin incubation. Scale bar: 200 μm.

Following the excellent antifouling performance against protein adsorption confirmed by DBCO-mPEG functionalization via SPAAC on the poly(Tyr-3-N_3_) films, we applied soft lithography to achieve spatially controlled protein patterning (Figure 3) [34]. For this purpose, we used the micromolding in capillaries (MIMIC) technique, which allows the site-selective immobilization of functional molecules by guiding the solutions through polydimethylsiloxane (PDMS) microchannels (50-µm wide, 100-µm spacing) [27]. Given the rapid kinetics of our surface reaction, it is expected to be compatible with MIMIC, enabling efficient regioselective mPEG immobilization. After flowing the DBCO-mPEG solution through the channels for 10 min, the PDMS channels were removed, and the surface was exposed to FITC-albumin. Consequently, fluorescence signals from the adhered FITC-albumin appeared exclusively in regions lacking mPEG, confirming protein patterning (Figure 3a). In contrast, the same MIMIC procedure on polydopamine substrates with DBCO-mPEG did not produce any patterns, confirming the absence of reactivity and serving as a control to confirm that the observed patterning on poly(Tyr-3-N_3_) films arose from SPAAC-based functionalization (Figure S4a).

In addition to the MIMIC demonstration, where functional molecules were introduced by solution flow through microchannels, the rapid surface reactivity enabled by SPAAC was also applied to microcontact printing (µCP), another soft lithography method that transfers ligands by direct contact from a patterned PDMS stamp [35]. Using a PDMS stamp having a grid pattern of 100-µm squares separated by 50-µm gaps, we first inked the stamp with DBCO-EG_4_-biotin (5 mM in EtOH) and then transferred it onto poly(Tyr-3-N_3_) films. After passivating the non-reacted regions by immersing the films in a DBCO-mPEG solution (1 mM in PBS) for 30 min, subsequent incubation with Alexa Fluor^TM^ 488 streptavidin produced a well-defined square fluorescence pattern arising from the bio-specific binding between the prepatterned biotin and streptavidin, thereby confirming effective regioselective immobilization (Figure 3b). In contrast, when the same µCP procedure was performed on polydopamine-coated surfaces, which lack azide functionalities, the resulting fluorescence signals appeared diffuse and irregular, unlike the well-defined square patterns observed for the poly(Tyr-3-N_3_) films (Figure S4b). This outcome can be attributed to nonspecific physical contact between the PDMS stamp and the substrate, rather than specific chemical ligation. Collectively, these results indicate that, unlike polydopamine films, the poly(Tyr-3-N_3_) films provide a reliable platform for rapid and spatially controlled protein patterning through SPAAC chemistry, as demonstrated by both MIMIC and µCP.

**Figure 4:**
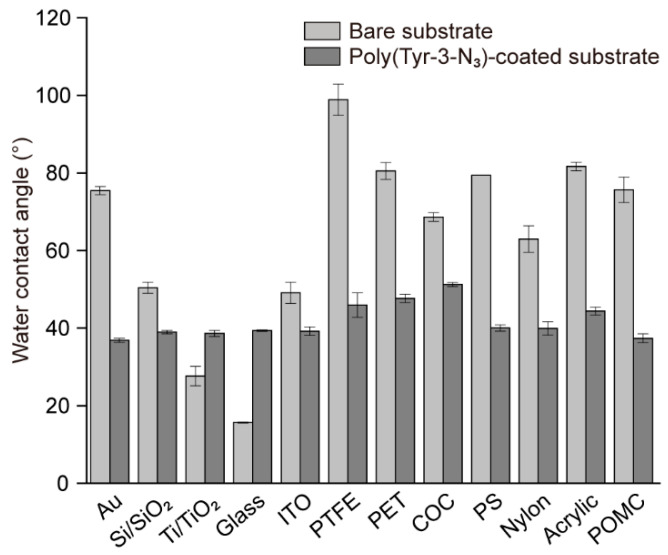
Comparison of water contact angles measured on various substrates before and after coating with poly(Tyr-3-N_3_) films.

In addition to enabling rapid surface functionalization and spatially controlled protein patterning, SPAAC allowed us to examine whether the optimized poly(Tyr-3-N_3_) coating strategy could be generalized to diverse substrates (Figure 4). The same coating protocol was applied to a series of inorganic and polymeric materials including gold (Au), silicon wafers (Si/SiO_2_), titanium dioxide (Ti/TiO_2_), glass, indium tin oxide (ITO), and various carbon- and plastic-based substrates such as polytetrafluoroethylene (PTFE), polyethylene terephthalate (PET), cyclic olefin copolymer (COC), polystyrene (PS), nylon, acrylic, and polyoxymethylene copolymer (POMC). In addition, the wettability of each surface was characterized using water contact angle (WCA) measurements before and after coating [36]. The pristine substrates had distinct WCA depending on their intrinsic surface physicochemical properties. However, after poly(Tyr-3-N_3_) deposition, all values converged to approximately 40°, demonstrating that the coating provided a common wettability independent of the underlying material. These results confirmed that poly(Tyr-3-N_3_) affords a substrate-independent platform with broad applicability.

**Figure 5:**
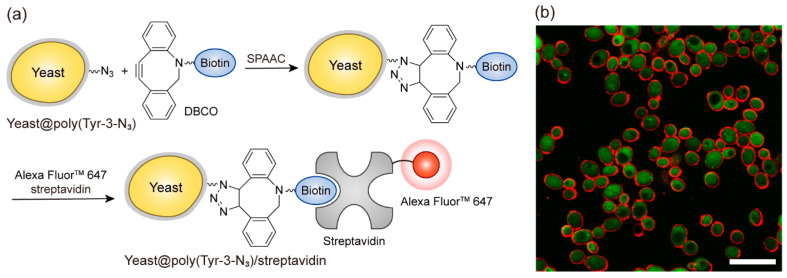
Cyto-compatible coating and functionalization of yeast cell surfaces with poly(Tyr-3-N_3_) films. (**a**) Schematic illustration of the coating and functionalization process. Yeast cells are encapsulated with a poly(Tyr-3-N_3_) layer, forming yeast@poly(Tyr-3-N_3_). The coated cells are then functionalized with DBCO-EG_4_-biotin, followed by bio-specific binding of Alexa Fluor^TM^ 647 streptavidin, yielding yeast@poly(Tyr-3-N_3_)/streptavidin. (**b**) Confocal laser-scanning microscopy (CLSM) images of yeast@poly(Tyr-3-N_3_)/streptavidin showing green intracellular fluorescence from fluorescein diacetate (FDA) in viable cells and red fluorescence from Alexa Fluor^TM^ 647 streptavidin at the cell periphery, confirming surface functionalization. Scale bar: 100 μm.

Because the coating is formed under physiological aqueous conditions (pH 7.4), we next examined whether it could be applied to living cell surfaces. This biocompatible coating approach is consistent with the concept of single-cell nanoencapsulation (SCNE), which involves the formation of thin artificial layers on individual cells to provide protective or functional properties while preserving their biological activity [37]. SCNE has been applied in cell-based sensors [38], biocatalysis [39], and probiotics [40], where cytocompatibility is critical. In this regard, we aimed to determine whether poly(Tyr-3-N_3_) films could be formed on living cells in a cyto-compatible manner and whether SPAAC could be harnessed to enable imaging of the cell surfaces (Figure 5). Yeast cells were selected as a representative eukaryotic model widely used in related studies, thereby facilitating comparisons with existing research [41]. Figure 5a illustrates the biocompatible coating and subsequent functionalization of yeast cells. Under the same conditions used for the planar substrates, the yeast was encapsulated in a poly(Tyr-3-N_3_) film, denoted yeast@poly(Tyr-3-N_3_). Biotin was then introduced via SPAAC with DBCO-EG_4_-biotin, and its presence was visualized through bio-specific interactions with Alexa Fluor^TM^ 647 streptavidin. The cells obtained in this manner are denoted yeast@poly(Tyr-3-N_3_)/streptavidin and used as a platform for the fluorescence imaging of the functionalized surface. To assess cytocompatibility, the relative viabilities of intact yeast, yeast@poly(Tyr-3-N_3_), and yeast@poly(Tyr-3-N_3_)/streptavidin were compared using a fluorescein diacetate (FDA) assay to measure intracellular esterase activity (Figure S5) [42]. Consequently, both yeast@poly(Tyr-3-N_3_) and yeast@poly(Tyr-3-N_3_)/streptavidin showed negligible differences in viability compared to intact yeast. In addition, confocal laser scanning microscopy (CLSM) revealed a distinct dual fluorescence signal: green emission from FDA in the viable cells and a red ring-shaped signal from Alexa Fluor^TM^ 647 streptavidin at the cell periphery (Figure 5b). Together, these results confirm that the developed coating system enables cyto-compatible cell surface engineering, highlighting its potential as a versatile platform for living cell surface modification.

## 4. Conclusions

In summary, we developed a melanin-inspired tyrosine–azide coating system optimized for SPAAC-based surface functionalization. By systematically varying the linker length and hydrophilicity, we identified Tyr-3-N_3_ as an optimized coating precursor that yielded stable azide-presenting films, enabling SPAAC functionalization under physiological conditions without film degradation. The resulting platform showed excellent antifouling performance against protein adsorption and allowed facile protein patterning through site-selective SPAAC immobilization using established soft lithography methods such as MIMIC and µCP. In addition, such coatings could be formed on diverse substrates, including living cell surfaces. Yeast encapsulation and subsequent metal-free SPAAC functionalization preserved cell viability and enabled clear visualization of the cell surfaces in a biocompatible manner, demonstrating its cytocompatibility. Collectively, this study introduces a substrate-independent and biocompatible coating platform that integrates melanin-mimetic film formation with SPAAC reactivity, offering broad potential for antifouling coatings [43], biosensing interfaces [44], and biocompatible cell surface modification [45].

## Data Availability

The original contributions presented in this study are included in the article/Supplementary Material. Further inquiries can be directed to the corresponding authors.

## Supplementary Materials

The following supporting information can be downloaded at: https://www.mdpi.com/article/10.3390/polym17222969/s1, including the synthesis of Tyr-1-N_3_, Tyr-2-N_3_, and Tyr-3-N_3_; the experimental method for the enzyme-linked immunosorbent assay (ELISA); yeast cell surface coating and functionalization procedures; and other supporting Figures S1–S5.

## References

[1] Baskin J.M., Prescher J.A., Laughlin S.T., Agard N.J., Chang P.V., Miller I.A., Lo A., Codelli J.A., Bertozzi C.R. (2007). Copper-free click chemistry for dynamic in vivo imaging. Proc. Natl. Acad. Sci. USA.

[2] Ning X., Guo J., Wolfert M.A., Boons G.-J. (2008). Visualizing metabolically labeled glycoconjugates of living cells by copper-free and fast Huisgen cycloadditions. Angew. Chem. Int. Ed..

[3] Debets M.F., van Berkel S.S., Schoffelen S., Rutjes F.P.J.T., van Hest J.C.M., van Delft F.L. (2010). Aza-dibenzocyclooctynes for fast and efficient enzyme PEGylation via copper-free (3+2) cycloaddition. Chem. Commun..

[4] Jewett J.C., Sletten E.M., Bertozzi C.R. (2010). Rapid Cu-free click chemistry with readily synthesized biarylazacyclooctynones. J. Am. Chem. Soc..

[5] Dommerholt J., Schmidt S., Temming R., Hendriks L.J.A., Rutjes F.P.J.T., van Hest J.C.M., Lefeber D.J., Friedl P., van Delft F.L. (2010). Readily accessible bicyclononynes for bioorthogonal labeling and three-dimensional imaging of living cells. Angew. Chem. Int. Ed..

[6] Cabrera-Quiñones N.C., López-Méndez L.J., Cruz-Hernández C., Guadarrama P. (2025). Click chemistry as an efficient toolbox for coupling sterically hindered molecular systems to obtain advanced materials for nanomedicine. Int. J. Mol. Sci..

[7] VanBrunt M.P., Shanebeck K., Caldwell Z., Johnson J., Thompson P., Martin T., Dong H., Li G., Xu H., D’Hooge F. (2015). Bowen and M. Marelli. Genetically encoded azide containing amino acid in mammalian cells enables site-specific antibody−drug conjugates using click cycloaddition chemistry. Bioconjug. Chem..

[8] Kuzmin A., Poloukhtine A., Wolfert M.A., Popik V.V. (2010). Surface functionalization using catalyst-free azide−alkyne cycloaddition. Bioconjug. Chem..

[9] Zeng D., Lee N.S., Liu Y., Zhou D., Dence C.S., Wooley K.L., Katzenellenbogen J.A., Welch M.J. (2012). ^64^Cu core-labeled nanoparticles with high specific activity via metal-free click chemistry. ACS Nano.

[10] DeForest C.A., Anseth K.S. (2012). Photoreversible patterning of biomolecules within click-based hydrogels. Angew. Chem. Int. Ed..

[11] Jonker A.M., Borrmann A., van Eck E.R.H., van Delft F.L., Löwik D.W.P.M., van Hest J.C.M. (2015). A fast and activatable cross-linking strategy for hydrogel formation. Adv. Mater..

[12] Han I., Kim S.Y., Hong S.-P., Choi I.S., Cho W.K. (2023). Functional surfaces with zwitterionic carboxybetaine L-3,4-dihydroxyphenylalanine: Synthesis, coating, and antifouling applications. Prog. Org. Coat..

[13] Sun F., Wu K., Hung H.-C., Zhang P., Che X., Smith J., Lin X., Li B., Jain P., Yu Q. (2017). Paper sensor coated with a poly(carboxybetaine)-multiple DOPA conjugate via dip-coating for biosensing in complex media. Anal. Chem..

[14] Kim B.J., Cho H., Park J.H., Mano J.F., Choi I.S. (2018). Strategic advances in formation of cell-in-shell structures: From syntheses to applications. Adv. Mater..

[15] Kim J.Y., Kim W.I., Youn W., Seo J., Kim B.J., Lee J.K., Choi I.S. (2018). Enzymatic film formation of nature-derived phenolic amines. Nanoscale.

[16] Lee H., Dellatore S.M., Miller W.M., Messersmith P.B. (2007). Mussel-inspired surface chemistry for multifunctional coatings. Science.

[17] Park S., Bisht H., Park S., Jeong J., Hong Y., Chu D., Koh M., Hong D. (2025). Melanin-inspired maleimide coatings on various substrates for rapid thiol functionalization. Macromol. Biosci..

[18] Jeong J., Bisht H., Park S., Hong Y., Shin G., Hong D. (2023). Formation of antifouling brushes on various substrates using a melanin-inspired initiator film. Langmuir.

[19] Hong Y., Kim B., Jeong J., Bisht H., Park S., Hong D. (2022). Antifouling surface coating on various substrates by inducing tyrosinase-mediated oxidation of a tyrosine-conjugated sulfobetaine derivative. Biomacromolecules.

[20] Bisht H., Hong Y., Park S., Hwang Y., Hong D. (2025). Fabrication of versatile antifouling coatings inspired by melanogenesis using a tyrosine-conjugated carboxybetaine derivative. Langmuir.

[21] Declas N., Maynard J.R.J., Menin L., Gasilova N., Götze S., Sprague J.L., Stallforth P., Matile S., Waser J. (2022). Tyrosine bioconjugation with hypervalent iodine. Chem. Sci..

[22] Madl C.M., Heilshorn S.C. (2017). Tyrosine-selective functionalization for bio-orthogonal cross-linking of engineered protein hydrogels. Bioconjug. Chem..

[23] Wang P., Silverman S.K. (2016). DNA-catalyzed introduction of azide at tyrosine for peptide modification. Angew. Chem. Int. Ed..

[24] Bisht H., Jeong J., Hong Y., Park S., Hong D. (2022). Development of universal and clickable film by mimicking melanogenesis: On-demand oxidation of tyrosine-based azido derivative by tyrosinase. Macromol. Rapid Commun..

[25] Hong S., Na Y.S., Choi S., Song I.T., Kim W.Y., Lee H. (2012). Non-covalent self-assembly and covalent polymerization co-contribute to polydopamine formation. Adv. Funct. Mater..

[26] Yang W., Liu C., Chen Y. (2018). Stability of polydopamine coatings on gold substrates inspected by surface plasmon resonance imaging. Langmuir.

[27] Kang S.M., You I., Cho W.K., Shon H.K., Lee T.G., Choi I.S., Karp J.M., Lee H. (2010). One-step modification of superhydrophobic surfaces by a mussel-inspired polymer coating. Angew. Chem. Int. Ed..

[28] Hong S., Wang Y., Park S.Y., Lee H. (2018). Progressive fuzzy cation-π assembly of biological catecholamines. Sci. Adv..

[29] Kim E., Koo H. (2019). Biomedical applications of copper-free click chemistry: In vitro, in vivo, and ex vivo. Chem. Sci..

[30] Ryu J.H., Messersmith P.B., Lee H. (2018). Polydopamine surface chemistry: A decade of discovery. ACS Appl. Mater. Interfaces.

[31] Lee B.S., Lee J.K., Kim W.-J., Jung Y.H., Sim S.J., Lee J., Choi I.S. (2007). Surface-initiated, atom transfer radical polymerization of oligo(ethylene glycol) methyl ether methacrylate and subsequent click chemistry for bioconjugation. Biomacromolecules.

[32] Al-Bataineh S.A., Luginbuehl R., Textor M., Yan M. (2009). Covalent immobilization of antibacterial furanones via photochemical activation of perfluorophenylazide. Langmuir.

[33] Hong J., Choi S., Jwa D.G., Kim M., Kang S.M. (2020). Mussel-inspired, one-step thiol functionalization of solid surfaces. Langmuir.

[34] Xia Y., Whitesides G.M. (1998). Soft lithography. Angew. Chem. Int. Ed..

[35] Rozkiewicz D.I., Jańczewski D., Verboom W., Ravoo B.J., Reinhoudt D.N. (2006). “Click” chemistry by microcontact printing. Angew. Chem. Int. Ed..

[36] Kang S.M., Rho J., Choi I.S., Messersmith P.B., Lee H. (2009). Norepinephrine: Material-independent, multifunctional surface modification reagent. J. Am. Chem. Soc..

[37] Lee H., Park J., Kim N., Youn W., Yun G., Han S.Y., Nguyen D.T., Choi I.S. (2022). Cell-in-catalytic-shell nanoarchitectonics: Catalytic empowerment of individual living cells by single-cell nanoencapsulation. Adv. Mater..

[38] Zamaleeva A.I., Sharipova I.R., Shamagsumova R.V., Ivanov A.N., Evtugyn G.A., Ishmuchametova D.G., Fakhrullin R.F. (2011). A whole-cell amperometric herbicide biosensor based on magnetically functionalised microalgae and screen-printed electrodes. Anal. Methods.

[39] Oh J., Kumari N., Kim D., Kumar A., Lee I.S. (2024). Ultrathin silica-tiling on living cells for chemobiotic catalysis. Nat. Commun..

[40] Han S.Y., Nguyen D.T., Kim B.J., Kim N., Kang E.K., Park J.H., Choi I.S. (2023). Cytoprotection of probiotic *Lactobacillus acidophilus* with artificial nanoshells of nature-derived eggshell membrane hydrolysates and coffee melanoidins in single-cell nanoencapsulation. Polymers.

[41] Wang B., Liu P., Jiang W., Pan H., Xu X., Tang R. (2008). Yeast cells with an artificial mineral shell: Protection and modification of living cells by biomimetic mineralization. Angew. Chem. Int. Ed..

[42] Yang S.H., Kang S.M., Lee K.-B., Chung T.D., Lee H., Choi I.S. (2011). Mussel-inspired encapsulation and functionalization of individual yeast cells. J. Am. Chem. Soc..

[43] Wang J., Li L., Wu Y., Liu Y. (2025). Design and application of antifouling bio-coatings. Polymers.

[44] Skigin P., Robin P., Kavand A., Mensi M., Gerber-Lemaire S. (2024). “Grafting-from” and “grafting-to” poly(N-isopropyl acrylamide) functionalization of glass for DNA biosensors with improved properties. Polymers.

[45] Rheem H.B., Kim N., Nguyen D.T., Baskoro G.A., Roh J.H., Lee J.K., Kim B.J., Choi I.S. (2025). Single-cell nanoencapsulation: Chemical synthesis of artificial cell-in-shell spores. Chem. Rev..

