# Supplementary material for: Linker-Engineered Tyrosine–Azide Coatings for Stable Strain-Promoted Azide–Alkyne Cycloaddition (SPAAC) Functionalization
Source: Polymers (Basel). 2025 Nov 7;17(22):2969. doi: 10.3390/polym17222969 (PMC12656541; doi:10.3390/polym17222969)
Supplement: Supplementary file 1 [file polymers-17-02969-s001.zip › polymers-3958445-supplementary.pdf]

Supporting Information

Linker-Engineered Tyrosine–Azide Coatings for Stable Strain-Promoted Azide–Alkyne Cycloaddition (SPAAC) Functionalization

Suho Park [1,2], Himani Bisht [1,2], Jiwoo Park [3], Seongchul Park [1,2], Yubin Hong [1,2], Daeun Chu [1,2], Minseob Koh [1,2], Hojae Lee [3,*] and Daewha Hong [1,2,*]

1 Department of Chemistry and Chemistry, Institute for Functional Materials, Pusan National University, Busan 46241, Republic of Korea; sohomish@pusan.ac.kr (S.P.); himani@pusan.ac.kr (H.B.); minseob.koh@pusan.ac.kr (M.K.)
2 Institute for Future Earth, Pusan National University, Busan 46241, Republic of Korea
3 Department of Chemistry, Hallym University, Chuncheon 24252, Republic of Korea
* Correspondence: hlee@hallym.ac.kr (H.L.); dw_hong@pusan.ac.kr (D.H.)

CONTENTS

Materials
Synthesis of Tyr-1-N_3
Synthesis of Tyr-2-N_3
Synthesis of Tyr-3-N_3
Enzyme-Linked Immunosorbent Assay (ELISA)
Yeast Cell Surface Coatings Using Tyr-3-N_3
Functionalization of Yeast@poly(Tyr-3-N_3) for Cell Surface Imaging
Characterization
Figure S1. Solubility and film formation of Tyr-1-N_3, Tyr-2-N_3, and Tyr-3-N_3.
Figure S2. Characterization of poly(Tyr-2-N_3) films before and after SPAAC functionalization with DBCO acid.
Figure S3. Relative fibrinogen adsorption on mPEG-modified surfaces, quantified by enzyme-linked immunosorbent assay (ELISA).
Figure S4. Control fluorescence-microscopy images obtained from soft lithography (MIMIC and μCP) with DBCO ligands on polydopamine substrates.
Figure S5. Cell viability measurement of intact yeast, yeast@poly(Tyr-3-N_3), and yeast@poly(Tyr-3-N_3)/streptavidin.

Materials

The following reagents were purchased from commercial suppliers and used as received: *N*-(*tert*-butoxycarbonyl)-*O*-*tert*-butyl-L-tyrosine (protected tyrosine, >98.0%, TCI), 1-(3-dimethylaminopropyl)-3-ethylcarbodiimide hydrochloride (EDC, >98.0%, Thermo Scientific), 4-dimethylaminopyridine (DMAP, >99.0%, TCI), hydrogen chloride (HCl, 4 N solution in 1,4-dioxane, Thermo Scientific), 11-azido-3,6,9-trioxaundecan-1-amine, (>90.0%, TCI), 10-azido-1-decanamine (95.0%, BLDpharm), 3-azidopropan-1-amine (98.0%, Enamine), sodium sulfate anhydrous (Na_2SO_4, >99.0%, Daejung), tyrosinase from mushrooms (≥1000 units mg^{-1}, Sigma-Aldrich), phosphate-buffered saline (PBS, Thermo Fisher), fibrinogen from human plasma (fibrinogen, 50–70% protein, Sigma), horseradish peroxidase (HRP)-conjugated anti-fibrinogen (Novus Biologicals), 3,3',5,5'-tetramethylbenzidine (TMB, 98%, Alfa Aesar), dimethyl sulfoxide (DMSO, >99.5%, Daejung), citric acid (>99%, Alfa Aesar), sodium phosphate dibasic (>99%, Sigma-Aldrich), hydrogen peroxide (35%, Alfa Aesar), sulfuric acid (H_2SO_4, 95.0–98.0%, Alfa Aesar), fluorescein diacetate (FDA, Sigma-Aldrich), Alexa Fluor™ 647 streptavidin (Invitrogen), sodium chloride (NaCl, ≥99.0%, Daejung), DBCO-EG_4-biotin (99%, Broad Pharm), and yeast-extract-peptone-dextrose broth (YPD broth, Duchefa Biochemie). Organic solvents, including dichloromethane (CH_2Cl_2, >99.5%), methanol (MeOH, >99.9%), diethyl ether (>99.0%), and acetone (≥99.5%) were purchased from Daejung. Deionized water was purified using a Millipore water purification system.

Synthesis of Tyr-1-N_3

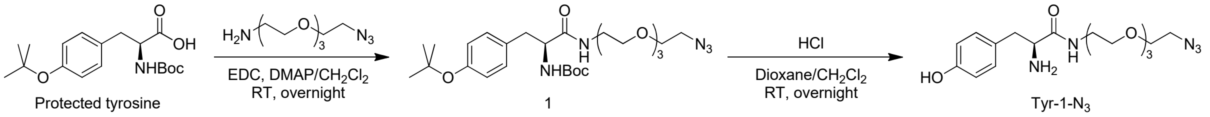

Tyr-1-N_3 was synthesized following our previously reported procedure (*Macromol. Rapid Commun.*, **2022**, *43*, 2200089) with slight modifications. To a solution of protected tyrosine (1.00 g, 2.96 mmol) in CH_2Cl_2 (20 mL) were added EDC (0.68 g, 3.55 mmol), DMAP (0.43 g, 3.52 mmol), and 11-azido-3,6,9-trioxaundecan-1-amine (0.71 g, 3.25 mmol) sequentially. The reaction mixture was stirred at room temperature (RT) overnight. The mixture was then washed with deionized water (3 × 50 mL) and brine (50 mL), dried over Na_2SO_4, and concentrated under reduced pressure. The crude product was purified by column chromatography (silica gel, CH_2Cl_2/MeOH, 95:5) to afford compound 1 (1.20 g) as a colorless sticky solid. Yield: 75%. ^1H NMR (400 MHz, $CDCl_3$): δ 7.08 (d, J = 8.5 Hz, 2H), 6.89 (d, J = 8.5 Hz, 2H), 6.28 (br, 1H), 5.10 (br, 1H), 4.28–4.26 (m, 1H), 3.67–3.36 (m, 16H), 3.00–2.97 (m, 2H), 1.39 (s, 9H), 1.31 (s, 9H). ^{13}C NMR (100 MHz, $CDCl_3$): δ 171.26, 155.24, 154.11, 131.62, 129.68, 124.10, 79.70, 78.22, 70.58, 70.48, 70.46, 70.16, 69.94, 69.49, 55.58, 50.56, 39.12, 38.21, 28.75, 28.22. HRMS (ESI) calculated for $C_{26}H_{44}N_5O_7$ $[M + H]^+$, 538.3235; found: 538.3239.

Compound 1 (1.12 g, 2.08 mmol) was dissolved in CH_2Cl_2 (10 mL). To this solution, 4 N HCl in dioxane (5.2 mL, 20.80 mmol) was added dropwise. The reaction mixture was stirred overnight at RT. The solvent was removed under reduced pressure, and the resulting crude product was dissolved in MeOH (1 mL). The solution was then slowly added dropwise into cold diethyl ether (20 mL) to induce precipitation, affording Tyr-1-N_3 salt (0.85 g) as a pale-yellow solid; mp 117 °C. Yield: 98% ^1H NMR (400 MHz, CD_3OD): δ 6.98 (d, J = 8.5 Hz, 2H), 6.68 (d, J = 8.5 Hz, 2H), 3.61–3.24 (m, 17H), 2.82–2.68 (m, 2H). ^{13}C NMR (100 MHz, CD_3OD): δ 169.68, 158.16, 131.65, 126.09, 116.74, 71.56, 71.52, 71.42, 71.20, 71.03, 70.17, 55.96, 51.71, 40.46, 37.88. HRMS (ESI) calculated for $C_{17}H_{28}N_5O_5$ $[M + H]^+$ 382.2085, found 382.2091.

Synthesis of Tyr-2-N_3

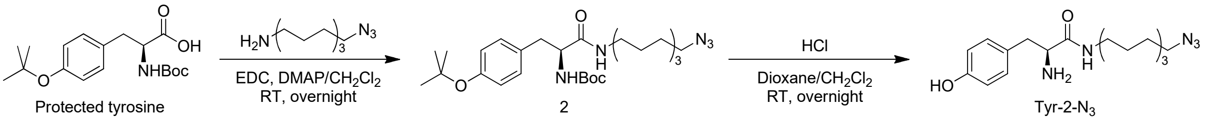

To a solution of protected tyrosine (1.00 g, 2.96 mmol) in CH_2Cl_2 (20 mL) were added EDC (0.68 g, 3.55 mmol), DMAP (0.43 g, 3.52 mmol), and 10-azido-1-decanamine (0.65 g, 3.27 mmol) sequentially. The reaction mixture was stirred at RT overnight. The mixture was then washed with deionized water (3 × 50 mL) and brine (50 mL), dried over Na_2SO_4, and concentrated under reduced pressure. The crude product was purified by column chromatography (silica gel, CH_2Cl_2/MeOH, 95:5) to afford compound 2 (1.26 g) as a pale-yellow solid. Yield: 82%. ^{1}H NMR (400 MHz, $CDCl_3$): δ 7.07 (d, J = 8.5 Hz, 2H), 6.88 (d, J = 8.5 Hz, 2H), 5.83 (br, 1H), 5.17 (br, 1H), 4.23–4.22 (m, 1H), 3.25–3.20 (m, 2H), 3.14–3.08 (m, 2H), 3.01–2.91 (m, 2H), 1.58–1.55 (m, 2H), 1.39 (s, 9H), 1.33–1.20 (m, 14H), 1.30 (s, 9H). ^{13}C NMR (100 MHz, $CDCl_3$): δ 171.15, 155.49, 154.33, 131.79, 129.81, 124.33, 124.27, 80.07, 78.42, 77.36 56.20, 51.54, 39.53, 38.30, 29.47, 29.44, 29.25, 29.18, 28.91, 28.38, 26.86, 26.77. HRMS (ESI) calculated for $C_{28}H_{48}N_5O_4$ $[M + H]^+$, 518.3701; found: 518.3707.

Compound 2 (1.20 g, 2.32 mmol) was dissolved in CH_2Cl_2 (10 mL). To this solution, 4 N HCl in dioxane (5.80 mL, 23.20 mmol) was added dropwise. The reaction mixture was stirred overnight at RT. The solvent was removed under reduced pressure, and the resulting crude product was dissolved in MeOH (1 mL). The solution was then slowly added dropwise into cold diethyl ether (20 mL) to induce precipitation, affording Tyr-2-N_3 salt (0.82 g) as a pale-yellow solid; mp 93 °C. Yield: 89%. ^{1}H NMR (400 MHz, CD_3OD): 7.01 (d, J = 8.5 Hz, 2H), 6.71 (d, J = 8.5 Hz, 2H), 3.49−3.47 (br, 1H), 3.29−3.25 (m, 2H), 3.18−3.03 (m, 2H), 2.85−2.76 (m, 2H), 1.60−1.55 (m, 2H), 1.40−1.18 (m, 14 H). ^{13}C NMR (100 MHz, CD_3OD): δ 176.15, 157.39, 131.38, 129.13, 116.28, 57.82, 52.45, 41.63, 40.31, 30.57, 30.53, 30.38, 30.30, 30.24, 29.91, 27.93, 27.82. HRMS (ESI) calculated for $C_{19}H_{32}N_5O_2$ $[M + H]^+$, 362.2551; found: 362.2553.

Synthesis of Tyr-3-N_3

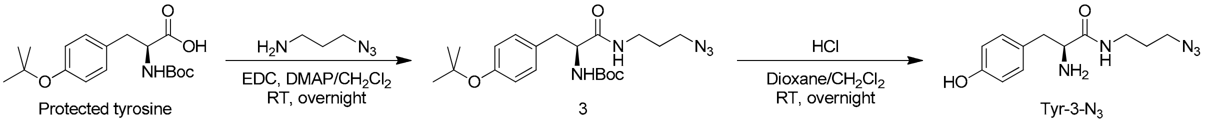

To a solution of protected tyrosine (1.00 g, 2.96 mmol) in CH_2Cl_2 (20 mL) were added EDC (0.68 g, 3.55 mmol), DMAP (0.43 g, 3.52 mmol), and 3-azido-1-propanamine (0.33 g, 3.30 mmol) sequentially. The reaction mixture was stirred at RT overnight. The mixture was then washed with deionized water (3 × 50 mL) and brine (50 mL), dried over Na_2SO_4, and concentrated under reduced pressure. The crude product was purified by column chromatography (silica gel, CH_2Cl_2/MeOH, 95:5) to afford compound 3 (1.10 g) as a pale-yellow solid. Yield: 88%. ^{1}H NMR (400 MHz, $CDCl_3$): δ 7.05 (d, J = 8.5 Hz, 2H), 6.86 (d, J = 8.5 Hz, 2H), 6.43 (br, 1H), 5.32−5.30 (br, 1H), 4.30−4.24 (m, 1H), 3.23−3.16 (m, 4H), 2.94−2.93 (m, 2H), 1.63−1.59 (m, 2H), 1.36 (s, 9H), 1.28 (s, 9H). ^{13}C NMR (100 MHz, $CDCl_3$): δ 171.71, 155.58, 154.27, 131.62, 129.75, 124.24, 80.05, 78.40, 56.09, 48.95, 38.09, 36.80, 28.82, 28.63, 28.31. HRMS (ESI) calculated for $C_{21}H_{34}N_5O_4$ $[M + H]^+$, 420.2605; found: 420.2606.

Compound 3 (1.03 g, 2.45 mmol) was dissolved in CH_2Cl_2 (10 mL). To this solution, 4 N HCl in dioxane (6.15 mL, 24.60 mmol) was added dropwise. The reaction mixture was stirred overnight at RT. The solvent was removed under reduced pressure, and the resulting crude product was dissolved in MeOH (1 mL). The solution was then slowly added dropwise into cold diethyl ether

(20 mL) to induce precipitation, affording Tyr-3-N_3 salt (0.72 g) as a pale-yellow solid; mp 90 °C. Yield: 98%. ^1H NMR (400 MHz, CD_3OD): δ 7.07 (d, J = 8.5 Hz, 2H), 6.77 (d, J = 8.5 Hz, 2H), 3.90–3.87 (m, 1H), 3.34–3.13 (m, 4H), 3.01–2.96 (m, 2H), 1.68–1.61 (m, 2H). ^{13}C NMR (100 MHz, CD_3OD): δ 176.34, 157.37, 131.38, 129.07, 116.34, 57.83, 49.78, 41.51, 37.44, 29.48. HRMS (ESI) calculated for $C_{12}H_{18}N_5O_2$ $[M + H]^+$, 264.1455; found: 264.1451.

Enzyme-Linked Immunosorbent Assay (ELISA)

ELISA was conducted to assess fibrinogen adsorption on uncoated and coated surfaces. Surfaces were first incubated in PBS for 1 hour, rinsed with PBS, and then immersed in a fibrinogen solution (1 mg mL^{-1} in PBS) for another hour. After washing twice with PBS, the samples were treated with HRP-conjugated anti-fibrinogen antibody (1 μg mL^{-1} in PBS) for 30 minutes and washed twice with PBS. The substrates were gently rinsed with deionized water and dried under a stream of air. To evaluate HRP enzymatic activity, a reaction solution was prepared by mixing 1 mL of TMB stock solution (1 mg mL^{-1} in DMSO), 9 mL of citrate-phosphate buffer (5 mM, pH 5.5), and 2 μL of hydrogen peroxide. Then, 150 μL of this solution was added to each substrate and incubated in the dark for 30 minutes. The reaction solution was collected and immediately quenched with one-fourth volume of 2 M H_2SO_4. Absorbance was measured at 453 nm using a UV-visible spectrophotometer.

Yeast Cell Surface Coatings Using Tyr-3-N_3

For the washing process of yeast cells, the cells were subjected to centrifugation at 1500 rpm to form a pellet, after which the supernatant was discarded. The pellet was then resuspended in a fresh aqueous solution. A grain of instant yeast was cultured in yeast-extract-peptone-dextrose (YPD) broth with gentle shaking at 33 °C for 30 h. After washing the cells with aqueous NaCl (0.15 mM) and PBS, the yeast cells were dispersed in a Tyr-3-N_3 solution (5 mL, 2 mM in PBS), and the cell suspension was transferred to a coating chamber (diameter: 3.5 cm; height: 1.75 cm). Subsequently, an aliquot (20 μL) of the tyrosinase stock solution (10 kU mL^{-1}) was added to the coating chamber, which was then placed in an incubator (30 °C, 140 rpm) to initiate coating of the cell surface. After 6 h, the resulting poly(Tyr-3-N_3)-coated yeast cells (yeast@poly(Tyr-3-N_3)) were washed with aqueous NaCl and dispersed in PBS for further functionalization. In addition, fluorescein diacetate (FDA) was dissolved in acetone at a concentration of 10 mg mL^{-1} to prepare a stock solution. An aliquot (2 μL) of this FDA stock solution was then mixed with the cell suspension (1 mL) and incubated for 15 min at 30 °C in the dark with shaking. The green fluorescence of the live cells was observed using confocal laser scanning microscopy (CLSM, IX83 Confocal Microscope, Olympus).

Functionalization of Yeast@poly(Tyr-3-N_3) for Cell Surface Imaging

The prepared yeast@poly(Tyr-3-N_3) was dispersed in an aqueous solution of DBCO-EG_4-biotin (1 mM in PBS) for 10 min. At intervals of 2 min, the cell suspension was pipetted to prevent sedimentation, thereby facilitating effective heterogeneous surface reactions. The yeast@poly(Tyr-3-N_3) cells functionalized with biotin were washed with aqueous NaCl and then resuspended in an Alexa Fluor™ 647 streptavidin solution (100 μg mL^{-1} in PBS containing 1% w/v BSA) to induce biotin–streptavidin binding for 30 min, resulting in yeast@poly(Tyr-3-N_3)/streptavidin. At intervals of 5 min, the cell suspension was pipetted to prevent sedimentation and promote effective heterogeneous surface reactions. After washing with a aqueous NaCl, circular red fluorescence resulting from the immobilization of Alexa Fluor™ 647 streptavidin was observed using CLSM (IX83 Confocal Microscope, Olympus). An FDA assay was performed after streptavidin treatment to detect additional green fluorescence in the cytoplasm.

Characterization

The thickness of the organic layer on the substrate was measured using a spectroscopic ellipsometer at 400–700 nm and using a 70° angle of incidence (Elli-SE, Ellipso Technology). The static water contact angles of the coated films were measured using a contact angle analyzer (Phoenix 300, Surface & Electro-Optics). Attenuated total reflectance-Fourier transform infrared (ATR-FTIR) spectroscopy was performed using a Nicolet iS20 FTIR spectrometer (Thermo Scientific) equipped with a Smart SAGA reflectance accessory.

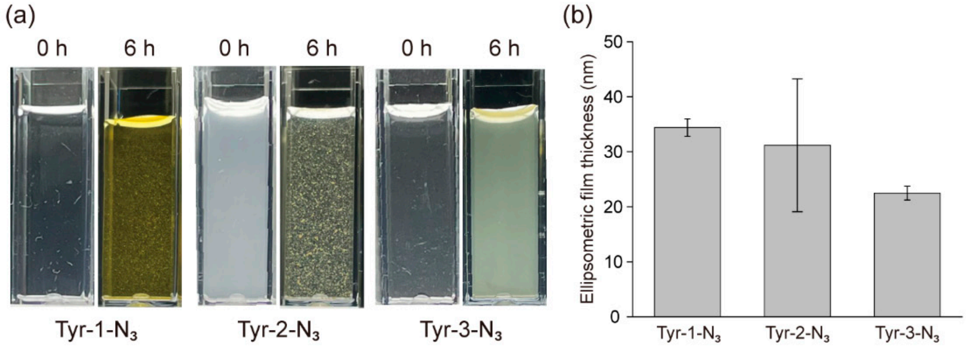

Figure S1. Solubility and film formation of Tyr-1-N_3, Tyr-2-N_3, and Tyr-3-N_3. (a) Photographs of precursor solutions in PBS before and after 6 h of tyrosinase treatment, highlighting the poor solubility and aggregation of Tyr-2-N_3. (b) Ellipsometry measurements of gold surfaces coated with films formed from Tyr-1-N_3, Tyr-2-N_3, and Tyr-3-N_3, revealing a markedly larger thickness deviation for the film derived from Tyr-2-N_3.

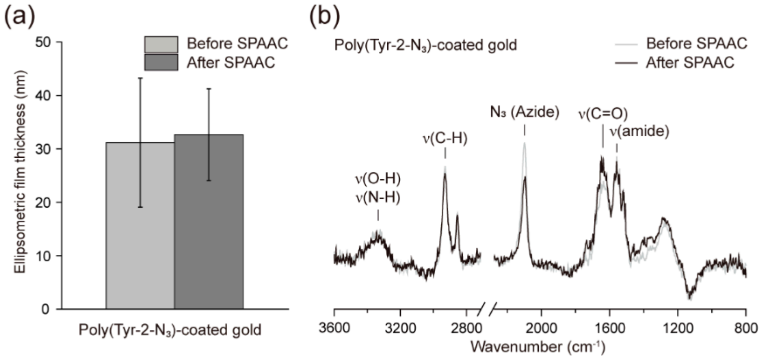

Figure S2. Characterization of poly(Tyr-2-N_3) films before and after SPAAC functionalization with DBCO acid. (a) Ellipsometric thickness of the coated films. (b) ATR-FTIR spectra of the gold surface.

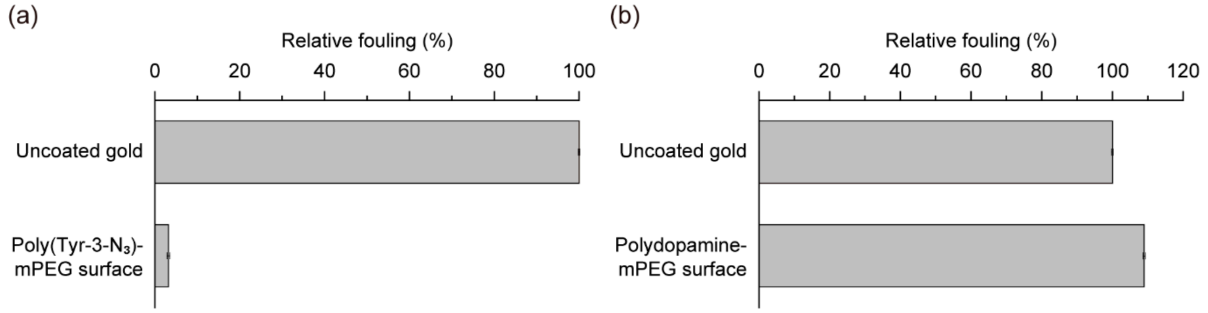

Figure S3. Relative fibrinogen adsorption on mPEG-modified surfaces, quantified by enzyme-linked immunosorbent assay (ELISA). (a) Comparison between uncoated gold and poly(Tyr-3-N_3)-mPEG surfaces. (b) Comparison between uncoated gold and polydopamine-mPEG surfaces. DBCO-mPEG and mPEG-thiol were used for functionalization of poly(Tyr-3-N_3) and polydopamine, respectively, under identical conditions (1 mM in PBS, 30 min).

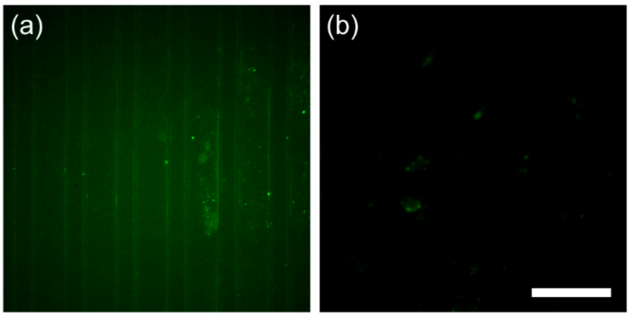

Figure S4. Control fluorescence-microscopy images obtained from soft lithography (MIMIC and μCP) with DBCO ligands on polydopamine substrates. (a) No protein patterns were observed when the MIMIC procedure was performed with DBCO-mPEG, consistent with the lack of SPAAC reactivity between polydopamine and DBCO. (b) The μCP procedure with DBCO-EG4-mPEG stamping produced only diffuse, irregular fluorescence signals, reflecting nonspecific physical contact between the PDMS stamp and the polydopamine surface rather than a specific chemical ligation. Scale bar: 200 μm.

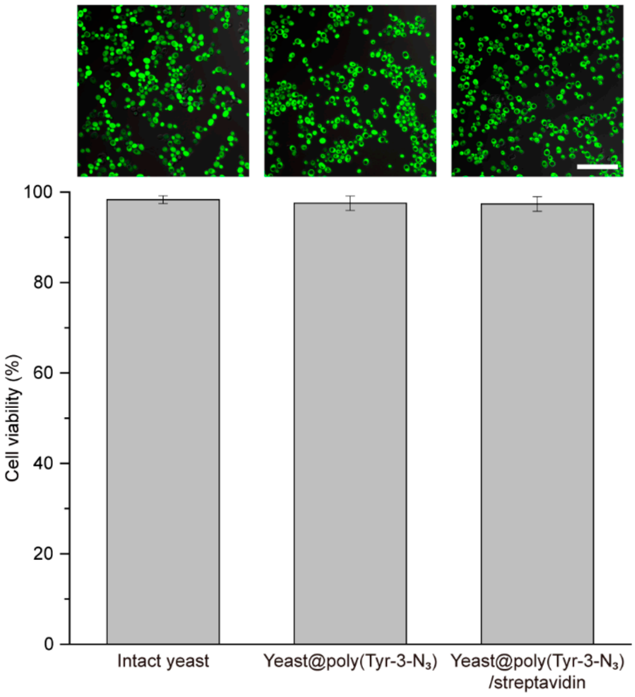

Figure S5. Cell viability measurement of intact yeast, yeast@poly(Tyr-3-N_3), and yeast@poly(Tyr-3-N_3)/streptavidin. Viability was assessed using the fluorescein diacetate (FDA) probe; green fluorescence indicates live cells. Scale bar: 50 μm.
